# Supplementary material for: Are carbon emissions trading and green financial instruments synergistic? -Comprehensive quantitative research based on content analysis
Source: PLoS One. 2024 Mar 7;19(3):e0298601. doi: 10.1371/journal.pone.0298601 (PMC10919601; doi:10.1371/journal.pone.0298601)
Supplement: S1 Data — (ZIP) [file pone.0298601.s001.zip › Data/地级市Z_F工作报告.zip!/║┌┴·╜¡/╞▀╠¿║╙╩╨/└┤╫╘╣1⁄2╓┌║┼í╛┬φ┐╦╩2╛▌═°í┐_2007.docx]

一、过去四年工作简要回顾

　　 八届人大一次会议以来，本届政府在省委、省政府和市委的正确领导下，团结和依靠全市人民，紧紧抓住老工业基地振兴等战略机遇，全面落实科学发展观，努力构建和谐社会，经济社会发展取得了显著成绩，是我市经济发展速度最快、质量最好、结构调整成效最明显的四年，是对外开放程度最高、社会事业进步最快、城市面貌变化最大的四年，是城市综合竞争力提升最快、人民群众得实惠最多、七台河发展进程中最辉煌的四年。

　　 经济综合实力连上新台阶。2006年全市生产总值实现114.2亿元，增长12.5%，年均增长12.7%；人均GDP达到12870元，位居全省第3位。规模以上工业增加值实现44.5亿元，增长13.5%，绝对值跃升到全省第5位，比2002年前移4位，年均增长23.8%。全口径财政收入实现16.3亿元，是2002年的3.7倍，年均增长39.5%；一般预算收入实现7.2亿元，是2002年的3.2倍，年均增长33.5%，是全省唯一财源建设先进市，勃利县连续四年被评为省财源建设先进县。GDP税收贡献率达到14.1 %，提高7.4个百分点。四年完成固定资产投资148.1亿元，年均增长23.1%。城镇居民人均可支配收入达到8908.7元，年均增长17%，农民人均纯收入3646元，年均增长9.1%。城乡居民人均储蓄达到11116元，年均增长14.3%。粮食产量突破10亿斤，畜牧业占农业增加值比重提高5个百分点，市级以上龙头企业发展到31家。原煤入洗能力由900万吨提高到2000万吨，焦炭产能由20万吨提高到400万吨，实现基本不卖原煤目标。非煤工业占全部工业比重提高8个百分点，双叶家具被评为中国名牌产品。民营经济迅速做大做强，销售收入超亿元、纳税超千万元企业从无到有发展到15户，亿达信公司年纳税超亿元、成为全省50强企业，宝泰隆等6户企业进入全省民营企业纳税50强。大商集团新玛特购物广场当年筹建当年投入使用，增强了城市辐射力。

　　 改革开放取得重大成果。在全省率先完成国有企业产权制度改革，赢得了加快发展的体制先机。市区财政分税制改革充分调动了区级抓发展的积极性，地方煤矿管理体制改革推进了安全管理重心下移，教育、人事、环保、城市执法等管理权相继下放到区，管理效能明显提高。行政审批制度改革四年精简审批项目1270项。农村税费改革从根本上减轻了农民负担。粮食流通体制改革促进了粮食购销市场化和经营主体多元化。七煤公司剥离办社会职能增强了企业竞争力。招商引资上项目成效显著。四年引进资金126亿元，实际利用外资2300万美元，新上项目1600个，投资亿元以上项目25个，千万元以上项目180个，香港环球公司、山东蓝裕集团、辽宁大商集团等知名企业落户我市。2006年外贸进出口总额实现1080万美元，增长142%，是2002年的7.7倍，年均增长66.6%。四年对上争取资金26.3亿元，大唐七台河发电厂二期和桃山水库二期项目开工建设，为我市长远发展奠定了坚实基础。棚户区改造规划得到省政府批复，七依高速公路完成前期工作。

　　 城乡基础设施日臻完善。累计投资51亿元，建设改造城市主要街路34条36万平方米，新建万宝、桃西立交桥和红卫、新兴大桥；建设改造供水管网282公里，解决了4.7万户居民吃水难问题；改造建设供热主管网32公里、换热站13座，增加供热面积317万平方米；建设煤气管线32公里和5万立方米储气柜，增加用户2.6万户；铺设排水管线35公里；新建扩建矿工广场等城市公园4个；房屋竣工面积375万平方米，一批高层和标志性建筑相继落成。欣源小区完成投资8亿元，新建住宅80万平方米1.1万户，8500户灾民喜迁新居，成为全省沉陷区治理样板工程。集中搬走电厂多年积存的两个灰渣山，取缔了域内所有小土焦，改善了生态环境。城市绿化、净化、美化、亮化水平全面提高。七密公路开工建设，七桦、七桃、七宝等16条公路竣工通车，东部城市群交通枢纽地位初步形成。建设通乡通村公路52条425.7公里，解决了89个村屯人畜饮水问题，有线电视通村率达到91%，农村生产生活条件明显改善。

　　 社会事业发展实现历史性跨越。四年投入4.1亿元，新建30所学校、总面积16.9万平方米，维修改造中小学危房21万平方米，城区学校基本实现楼房化，农村学校消灭了明火炉取暖，教育教学质量稳步提高。七台河职业学院填补了我市全日制高等教育空白。新建改造市医院门诊综合楼、传染病区、市县两级疾控中心、乡镇卫生院等一批医疗卫生项目，初步建成公共医疗卫生服务体系。新建殡仪馆、殡仪服务中心和莲花山公墓。竞技体育获全国冰上项目贡献奖和省政府突出贡献奖，王濛获都灵冬奥会金牌。科技服务经济发展能力逐年增强。广播电视和文化事业繁荣发展。民主法制和精神文明建设取得新成果。民族、宗教、物价、审计、旅游、计生、外事、侨务、人事、人防、老龄、残疾人事业等工作都取得了新的成绩。

　　 和谐社会建设扎实推进。四年实现新就业6.3万人，城镇登记失业率低于省控指标0.9个百分点，受到省和国家的表彰。完成193户原国有企业3.8万名职工并轨工作，筹集发放并轨资金2.62亿元。在全省率先实现原国有、集体企业职工全部纳入社会保障，率先实现关停破产和改制企业退休职工纳入基本医疗保险，率先解决退休职工项目外工资问题。城镇低保实现应保尽保，农村低保全面启动，新型合作医疗完成试点。在全省率先规范了行政工资津贴标准。建立了覆盖城乡的困难群众救助体系，各级财政发放困难学生救助资金294万元，救助困难学生1.6万人次，为城乡特困户和受灾群众发放钱物累计1.63亿元，提供法律援助318件次。市县社会福利院和老年公寓投入使用，新兴区、茄子河区社会福利院开工建设。平安七台河建设扎实推进，社会治安综合治理成效显著。以煤矿为重点的安全生产工作全面加强，地方煤矿安全质量标准化水平逐年提高，交通、消防等事故指标稳步下降，连续25年无森林火灾。妥善处理了大量经济、建设、改革等遗留问题，集中解决了一批疑难信访案件，进京、进省、到市访和集体越级访逐年减少。军政军民关系进一步密切，被评为省级双拥模范城。

　　 政府执政能力全面提高。政府职能转变取得重大进展。各级政府及职能部门服务企业和群众的意识明显增强，制约企业生产和影响群众生活的问题得到较好解决。依法行政水平明显提高。健全完善了政府工作制度和重大事项议事规则，决策科学化、民主化和法制化进程加快，人大代表建议、议案和政协委员提案得到及时有效办理。政府管理创新扎实推进。审批制度改革优化了经济发展环境，管理体制创新提高了行政效能，电子政务建设提升了办公自动化水平。加大了公务员选拔、培训和交流力度，公务员队伍整体素质明显增强，抓发展、抓落实的能力进一步提高。全面落实政务公开、政府采购、经营性土地招拍挂出让、建筑工程招投标等制度，政府廉政建设取得新成果。

　　 各位代表，四年来全市经济社会发展取得的显著成绩，是省委、省政府和市委正确领导的结果，是人大、政协正确监督和大力支持的结果，是驻七中省直部门密切配合、特别是市矿共建的结果，是全市人民齐心协力、干事创业的结果。在此，我代表市人民政府，向各位代表和全市人民表示衷心的感谢！

　　 过去四年工作中，我们积累了一些对今后发展具有指导意义的经验和启示。一是始终坚持以科学发展观为统领，不断深化对市情的认识，根据不同时期国家宏观政策，制定、坚持和完善了符合七台河实际的发展思路。二是始终坚持以发展为第一要务，善于在宏观经济走势中发现和把握机遇，依托资源优势，谋划建设了一批煤焦电化项目，抢占了市场先机，培植了新的立市产业。三是始终坚持以人为本，把关系群众切身利益的问题放在首位，集中财力优先解决社会保障、城市基础设施和社会事业等群众所想所盼的大事、难事。四是始终坚持用改革的办法解决发展中的问题，大胆进行体制机制创新，率先完成了地方国有企业产权制度、市区财政分税制、地方煤矿管理体制等重大改革，增强了经济社会发展活力。五是始终坚持招商引资和创建优良发展环境不放松，注重运用财政手段等政府资源引导调控市域经济发展，有效激活了以民营为主体的各类资本投资上项目。

　　 在充分肯定成绩的同时，必须正视资源型城市所面临的困难和问题。一是产业结构单一，接续产业规模较小，结构性矛盾突出，城市转型任务重。二是产业层次低，高新技术产业少，粗放的经济增长方式与资源接续和环境保护的矛盾突出，可持续发展压力大。三是城市基础设施和服务功能不够完善，综合承载能力不强，农村基础设施建设滞后，城区之间、城乡之间差距较大。四是政府职能转变还不完全适应市场经济要求，管理和服务水平亟待提高。五是关系群众切身利益的就业和社会保障、安全生产、社会公平，特别是困难群众生活等方面的问题还不少，构建和谐社会任重而道远。

　　 二、今后五年的基本构想

　　 新一届政府执政的五年，是我市加快资源型城市转型、实现建设龙江强市目标的重要时期。站在新的历史起点上，我们既面临着难得的机遇，也面临新的挑战。发达国家和国内发达地区产业转移加快，国家支持老工业基地振兴、资源型城市转型等政策深入实施，我省建设东部煤电化基地战略全面推进，加之我市多年形成的产业基础和发展后劲，都将给我市带来更加广阔的发展空间。另一方面，国际国内竞争更加激烈，周边城市竞相发展，我市经济正处于高速增长后的稳定发展期，加之煤炭产业链延伸项目投资大、准入条件高，非煤产业竞争激烈、对技术和人才依赖性强，新的经济增长因素不突出，加快发展的任务十分繁重。面对这种形势，我们必须统一思想，增强紧迫感，齐心协力谋发展，扎扎实实打基础，推动经济尽快实现新一轮高速增长。

　　 今后五年政府工作的总体要求是：以邓小平理论和“三个代表”重要思想为指导，全面落实科学发展观，坚持质量速度效益并重，强力推进煤炭循环经济，加快发展非煤接续产业，推动经济又好又快发展；加强生态建设和环境保护，打造国家园林城市；深化改革开放，增强经济社会发展活力；推进政府职能转变和管理创新，提高社会管理和公共服务水平；高度关注民生，加快构建和谐七台河，努力实现建设龙江强市的奋斗目标。

　　 按照这一要求，今后五年的奋斗目标是：煤焦电化四大基地进一步做强，煤炭循环经济框架基本形成；五大接续产业集群不断壮大，资源型城市转型迈出实质性步伐；现代农业初具规模，新农村建设取得重大成果；城市基础设施和生态环境建设成效明显，建成国家园林城市；社会事业全面进步，公共服务水平明显提高；就业再就业服务体系更加健全，社会保障和困难群众救助实现全覆盖；城乡居民收入大幅增长，人民生活水平显著提高；主要经济指标和人均指标进入全省前列，城市综合竞争力进一步提高，龙江强市建设取得重大进展。

　　 预期到2011年，本地生产总值力争实现220亿元，年均增长12％以上；全口径财政收入力争实现30亿元，年均增长13％以上；固定资产投资累计完成300亿元，年均增长20％以上；城镇居民人均可支配收入实现12500元，年均增长9.3％以上；农民人均纯收入实现5000元，年均增长6.5％以上。

实现上述目标，必须突出以下保证措施。

　　 ——坚定不移地实施科学发展战略。更加注重转变经济增长方式和提高增长质量，坚持走新型工业化道路，大力延伸煤炭循环经济链条，全面提高产业层次，保证经济又好又快发展。

　　 ——坚定不移地实施统筹发展战略。更加注重促进城乡之间、区域之间、产业之间、经济社会之间协调发展。扎实推进社会主义新农村建设，全面改善农村生产生活条件；支持县域、区域经济发展，壮大市域经济整体实力；大力发展现代服务业，满足群众生产生活需要；加快社会事业发展，提高公共服务水平。

　　 ——坚定不移地实施创新发展战略。更加注重改革开放和科技创新，全面深化各个领域的改革，创造发展的体制机制优势；提高企业自主创新能力，增强参与国际国内竞争实力；加强政府管理创新，营造招商引资和各类经济主体竞相发展的优良环境。

　　 ——坚定不移地实施可持续发展战略。更加注重节能降耗和环境保护，建设资源节约型和环境友好型社会；大力发展接续产业，加快资源型城市转型，增强可持续发展能力。

　　 ——坚定不移地实施和谐发展战略。更加注重以人为本，健全覆盖城乡的就业、社保、困难群众救助体系，让发展成果惠及全体群众；高度重视社会治安和安全生产，促进社会和谐稳定。

　　 三、2007年重点工作

　　 今年是新一届政府工作的开局之年，做好全年工作对于实现未来五年发展目标意义重大。2007年经济社会发展主要预期目标是：本地生产总值增长11％以上，财政一般预算收入增长10％以上，固定资产投资增长20％以上，城镇居民人均可支配收入增长9％，农民人均纯收入增长6.5％，万元GDP能耗下降4%。重点做好十项工作。

　　 （一）大力发展循环经济，加快新型工业化进程。

把工业作为推动全市经济尽快进入新一轮高速增长的关键，突出抓工业，重点抓项目，推进规模上企业进一步做大做强，扶持成长性好的中小企业加快发展。一是稳步发展原煤生产。支持符合安全生产条件的煤矿正常生产。加快58处改造井、48处在建井建设进度，新增产能150万吨。地方原煤产量力争达到800万吨，全市原煤产量稳定在2000万吨以上。二是努力提高焦炭产量。推进龙洋、鲁龙两个焦化项目尽快投产，新增产量50万吨。为周边市县原煤进入我市提供绿色通道，组合域外煤源保证焦化企业原料供应。经委要派专项小组驻省经委和铁路部门，协调解决铁路运输瓶颈制约难题。全市焦炭产量争取达到300万吨。三是加快煤化工产业发展。继续实行领导和部门项目包保责任制，帮助解决项目建设中的实际问题，加快宝泰隆、亿达信两个甲醇项目建设，保证年内投产，实现产量5万吨；推进圣迈公司10万吨焦油加工、博兴化工16万吨尿素、兴盛化工石墨电极等项目建设，力争年内投产。四是强力推进电力项目建设。大唐七台河发电厂二期工程力争完成投资17亿元，明年投产发电。宝泰隆10万千瓦热电联产项目力争年底投产。帮助德利能源做好10万千瓦热电联产项目前期工作。积极与大型电力企业联系，谋划建设风能、秸秆生物质等发电项目。五是倾斜支持非煤替代产业发展。支持双叶公司引进国外技术设备和国内异型玻璃、细木工板等配套企业，力争销售收入突破3亿元。引导双叶公司扩大与市内木制品加工企业的协作配套，带动域内木制品业实现集群发展。按照国家和省政策规定，制定扶持新型建材业发展政策，加大强制推广力度，保证宏源矸石砖等企业满负荷生产。加快北方建材2亿块矸石砖项目建设进度，力争年内投产。扶持勃农公司、七台河制药厂、康威药业等中小企业加快发展。

　　 （二）加快发展现代农业，扎实推进社会主义新农村建设。

按照省市新农村建设规划，以发展现代农业为重点，继续实行对农业的补贴政策，努力促进农业增效和农民增收。一是进一步优化种植业结构。推广农业先进适用技术，加大农机装备投入，普及优良品种，今年优质水稻、高油大豆、专用玉米面积分别达到25万亩、40万亩、20万亩。大力发展烤烟、北药、万寿菊、白瓜籽等特色经济作物，引导龙头企业通过与农户签订单、免费提供技术指导、及时兑付收购资金等措施，调动农民种植经济作物积极性。今年特色经济作物面积争取达到45万亩。增加无公害蔬菜面积，扩大基地规模，力争出口有所突破。今年新增棚室520栋、410亩。二是继续推进主辅换位进程。按照绿色畜产品标准，重点发展肉鸡、生猪规模化和标准化养殖，肉鸡、生猪出栏力争达到1000万只和50万头，新增大型养殖示范场50个、标准化小区6个。三是提高农业产业化水平。扶持龙头企业扩大规模，增加品种，提高带动能力。加快龙湘烟叶技术改造，支持大森林、蓝裕集团养殖基地建设，扶持林泓米业、金鼎大鹅、天龙浩等企业做强做大。推进日本味噌、韩国蔬菜等新项目建设。四是大力发展劳务经济。把就业技能培训作为促进农民增收的重要工作，继续实施“阳光培训”工程，新建15个农村劳动力培训基地，争取形成一两个特色品牌。全年转移农村劳动力4万人以上，实现收入2.35亿元。五是加强农村基础设施建设。继续实施新农村建设包保责任制，加大试点乡村扶持力度，抓两头带中间。通过财政支持、对上争取、村企共建、帮建单位资助、农民投资投劳等多种渠道，加大对农村基础设施投入。新建通村公路59条180公里，解决20个村8000人饮水问题，新建沼气池1100个，推广秸秆汽化炉1000个，改造中低产田2.8万亩，打农田补水井60个，治理万宝等8个小流域，建设小型水利设施30个。

　　 （三）积极发展现代服务业，推进第三产业提档升级。

坚持扩大总量、优化结构、提高水平、拓宽领域并重，制定出台扶持第三产业发展的优惠政策，巩固提升传统服务业，加快发展现代服务业。一是发展壮大物流业。抓好新兴区物流中心二期扩建，保证9月份投入使用。大力培植第三方物流业，引导加工企业与运输、仓储、配送企业联合，减少加工企业资金占用，降低流通环节成本。二是进一步完善市场载体。加大勃利农机大市场宣传力度，引进名牌产品经销商，扩大辐射范围。加快新兴区矿山配件和建材大市场建设，保证年内投入使用。建设城乡各类集贸市场，方便群众生产生活。以大商新玛特等大型商业企业为龙头，对“万村千乡市场工程”网点实行货物统一配送，通过对上争取和财政补贴，实现市区乡村全覆盖。三是积极发展旅游业。抓住桃山水库二期工程开工和七密公路通车机遇，大力开发旅游产业。启动沿湖观光大道建设，把桃山水库周边建成市民休闲度假区。提高西大圈、龙山森林公园基础设施建设水平和接待能力，发展旅游中介，开发旅游市场，融入全省东部旅游圈。四是努力做大外经贸业。改变我市没有边贸口岸、外经贸难有大发展的传统观念，抓住我省实施对俄对韩经贸战略升级机遇，壮大外经贸主体。扶持宏泰松果公司择址新建，支持双叶家具、金荞格尔金等外向型企业扩大出口规模。鼓励扶持有条件的企业到俄罗斯开展建筑工程承包、森林采伐、种植养殖等合作。五是大力发展社区服务业。充分发挥社区组织作用，制定社区服务业发展规划，有计划地推进社区服务基础设施建设。鼓励支持个人创办、领办家政服务、托老托幼、体育健身、卫生保健等社区服务业。六是促进信息、保险、证券业健康发展。创造诚信环境，支持信息、保险等行业改进服务、创新产品，更好地服务经济发展和群众生活。

　　 （四）继续抓好招商引资上项目和改善发展环境，努力增强经济发展后劲。

始终把招商引资上项目作为推动经济又好又快发展的重要手段，加强对招商引资规律的研究，创新引资途径和方式方法，培养壮大专业招商队伍，实现招商引资新的突破。一是更好地发挥企业招商主体作用。引导企业转变观念、放宽眼界，树立做强做大意识，主动与国内外大企业、大集团合资合作。帮助有引资意愿的企业提供信息、寻找伙伴、联系对接，力争企业自主引进项目有较大突破。二是积极承接发达地区产业转移。经济综合部门要深入研究发达地区产业转移动向，搜集产业转移项目信息，选择一批熟悉经济工作、有招商经验的干部组成专题小组，寻找有转移意向的企业上门对接，联系洽谈，力争引进一批规模较大的项目。三是做好项目储备工作。下功夫解决储备项目少、项目小、项目深度不够、吸引力不强等问题。加大各级政府项目前期投入，经济综合部门要组织专人研究项目，鼓励企业购买科研院所专利项目和吸引专利入股，建立起符合我市经济发展实际的项目库。四是加大园区建设投入。利用好国家批准的七台河经济开发区有关政策，加快金河等工业园区和七桃路、依宝路工业走廊规划建设。市区县要形成合力，加大对园区基础设施的投入，储备一定数量土地，真正建成招商引资上项目的载体。五是进一步搞好环境创建。围绕企业和群众反映集中的审批环节多、办事效率低、服务质量差、勒卡行为时有发生等问题，结合开展政务公开，进一步规范行政审批。凡是有审批职能的部门都要设立综合服务窗口，公开办理程序和时限，一个窗口对外，规范收费，限时办结。强化一站式办公中心服务功能，赋予派驻人员一定的审批权限，符合条件的事项当即批准。整顿涉及企业的各类收费和处罚，所有收费标准必须由物价、财政、审计等部门核定，规范收费部门及人员的行为和权力。认真研究国家政策规定，学习借鉴发达地区做法，修订完善招商引资优惠政策。根据投资规模，对新建项目给予基础设施建设、贷款贴息等资金支持，减免一定比例和年限的地方所得税收，免收全部行政性收费，自收自支单位服务费按成本收取。六是继续加大对上争取力度。加强与上级主管部门沟通联系，及时掌握国家和省重点投资方向。今年重点围绕新农村建设、发展循环经济、城乡基础设施、棚户区改造、矿山环境治理等项目，做实做细对上争取基础工作。加大已争取到项目的推进落实力度，把上级支持的工作做出最好的效果。

　　 （五）加强财税金融工作，提高服务经济社会发展的能力。

一是继续强化税收征管。做实纳税人信息采编等基础工作，推广地方煤矿税控经验，提高税收管控工作信息化水平，健全重点税源监控体系。组合财政、审计、税务稽查等部门力量，开展经常性的征税情况检查。支持税务部门履行职责，加强公安、司法机关与税务部门的配合，严厉打击偷、逃、骗税等违法犯罪行为。规范各类非税收入征收管理，合理确定非税收入指标。二是进一步优化支出结构。牢固树立过紧日子和艰苦奋斗思想，严格控购商品审批，大力压缩会议费、招待费、考察费等一般性支出。强化国有资产监管，推进政府投资评审工作，加强财政专项资金使用效能监督。财政资金重点向支持经济发展倾斜，向新农村建设倾斜，向发展社会事业倾斜，向社会保障特别是困难群众倾斜。巩固财政管理体制改革成果，依据事权合理划分市区财权，指导监督区级财政提高管理水平。三是支持金融部门健康发展。引导协调金融部门适应新形势，按照商业银行的运行机制，拓宽服务领域，创新金融产品，加大对地方经济特别是中小企业的支持力度。推进诚信七台河建设，启动建立企业和个人信用档案工作，为金融部门健康发展创造良好条件。

　　 （六）进一步提高城市建设管理水平，加快国家园林城市建设步伐。

坚持人与自然和谐相处，发展经济与保护环境并重，努力建设功能完善、布局合理、生态良好的山水园林城市。

　　 第一，继续提升城市功能。在完善中心区服务功能的同时，基础设施建设重点向新兴区和茄子河区倾斜，通过给政策、给支持，尽快改变新兴和茄子河区基础设施落后状况。今年重点搞好八个方面的建设。一是路桥工程。加快七密公路建设，确保9月末竣工通车。争取七依高速公路今年开工。完成七桦路扩建工程。改造新建学府街等城市道路，建设倭肯河北山大桥。二是供水改造。加快老化自来水管网改造，降低漏失率，扩大全天供水覆盖面。三是供热改造。完成热源二期工程建设，新增供热能力200万平方米，推进陈旧管网和单户单供改造，提高供热质量。四是污水处理。抓住大唐七台河电厂二期工程开工建设机遇，引资建设污水处理厂，并与电厂二期同步建设中水利用工程。五是垃圾处理。建设新兴区垃圾处理场，提高垃圾中转运输能力。解决民办环卫运营难题，扶持民办环卫健康发展。六是民用燃气。支持七煤公司完善煤气管网，新增用户4000户。七是沉陷区治理和棚户区改造。完成沉陷区治理投资2.7亿元，新建住宅23万平方米，安置灾民6000户。启动棚户区改造工程。八是排水工程。规划建设新兴区排水明渠和强排站，解决内涝问题；启动茄子河区东安街等排水主管网建设。第二，进一步提高城市管理水平。依据城市总体规划，落实分级管理责任，维护城市规划权威。依法加强城市管理，确保执法规范，群众满意。改进城市管理部门的服务，提高公用事业管理水平，满足群众生产生活需求。加快新兴区治理改造和茄子河区开发建设步伐，提高两区城市建设和管理水平。按照城市绿地系统规划，搞好城市道路、居民区、单位及园林、广场绿化；坚持政府组织和市场化运作相结合，完善城区保洁和清冰雪等公共设施管理的有效机制，进一步提高城市绿化、美化、净化水平。第三，强力推进环境保护和污染治理。高度重视环保工作，确保不欠新账，逐年还清欠账。今年重点打好三个攻坚战。一是空气污染治理攻坚战。严格执行国家环保政策，提高项目准入门槛，坚决取缔五小企业。严格控制排污总量，加大对企业污染排放监管力度，细化环保部门和区县政府的监管责任，落实企业环保投入的主体责任，新建项目达不到环保“三同时”的坚决不允许开工生产，污染排放超标的企业坚决停产整顿。大力推广使用型煤等清洁能源，中心区彻底取消散烧锅炉，城区服务业禁止原煤散烧，确保空气质量明显好转，群众满意度明显提高。二是水源地保护攻坚战。积极争取省环保、水利、国土资源等部门的支持配合，推进水库两岸山体耕地退耕还林；坚决取缔污染水源的砖厂、饭店及违章建筑；严密监管水库上游金矿、煤矿等工业污染物排放，保障城市饮水安全。 三是矿山环境恢复治理攻坚战。严格禁止影响城市环境和形象的开山采石、河道采沙等行为，搞好倭肯河沿岸、城市周边山体、废弃矿山的恢复治理和绿化。积极争取省森工、林业等部门支持，加快茄子河宏伟、岚峰等地区超坡度山地退耕还林、封山育林和植树造林，逐步恢复矿山生态环境。

　　 （七）坚持统筹协调发展，推进社会事业全面进步。

　　 把满足群众公共服务需求作为政府工作的重要职责，更加注重提高公共服务水平和促进公共服务均等化。一是优先发展教育。巩固“两基”成果，推进“双高普九”工作。扩大高中和职业教育规模，提高职业学院办学水平。进一步优化城乡学校网点布局，加强区属学校等薄弱学校建设和管理，改善农村办学条件，促进教育公平和均衡发展。加强校长、教师队伍和师德师风建设，全面提高教学质量，办人民满意教育。继续增加教育投入，建设职教中心、九小、越秀等一批学校教学楼，改造C、B级危房，加强新老三室建设。二是全面提高医疗卫生服务水平。完善医疗卫生网络建设，筹划建设市妇幼保健院，完成中医院改造，提高乡镇卫生院装备水平，健全社区医疗卫生服务体系，逐步改造村办卫生所。规范药品招标采购，推广单病种限价、药品价格公示等制度，探索解决看病贵问题。启动三区农村新型合作医疗。三是加快发展科技、文化、体育等社会事业。加快技术创新体系建设，发挥科技孵化器和科技企业投融资平台作用，支持大企业技术改造和中小科技企业发展。增加文化事业投入，鼓励支持文艺工作者创作更多宣传我市的优秀作品。支持报业发展。筹建文化、体育、老干部和青少年四个中心。稳定低生育水平，提高人口素质。加快有线电视数字化进程，提高广播电视节目质量。四是加快推进人才兴市进程。积极培养、引进各类专业技术人才，重点帮助企业解决人才短缺问题。制定优惠政策，解决优秀人才住房、子女就学等问题，以优厚待遇留住人才，创造条件用好人才。继续搞好公务员、企业家和事业单位技术人员培训。五是进一步加强精神文明和民主法制建设。深入贯彻《公民道德建设实施纲要》，广泛开展“八荣八耻”教育。扎实开展群众性精神文明创建活动，树立精神文明先进典型。推进“五五”普法工作，加快依法治市进程。做好兵役和民兵工作，落实优抚政策，推进双拥共建，争创全国双拥模范城。

　　 （八）高度关注民生，保证发展成果惠及全体群众。

　　 坚持以人为本，更加关注困难群体和重视改善群众生活， 全力解决好群众最关心最直接最现实的利益问题。

　　 第一，健全就业再就业服务体系。继续落实促进就业的各项政策，鼓励扶持自主创业，有针对性地搞好就业技能培训，注重培育特色品牌。加强择业观念教育，继续推进“三进三送”就业行动，搞好企业用工需求与劳动力求职的对接。全年实现新就业8000 人，城镇登记失业率低于4.5%。严格执行最低工资制度，监管企业按时足额发放职工特别是农民工工资，维护劳动者合法权益。第二，完善社会保障体系。加大社会保险扩面和保费征缴力度，加强社保基金管理，做到应收尽收、支付合理、管理严格、监督有力。严格执行最低生活保障政策，做到应保尽保、进出有序、良性循环。第三，建立覆盖城乡的困难群众救助体系。形成政府关心、单位资助、全社会关注困难群体的浓厚氛围。今年重点为困难群众办好十件实事。一是全部免收农村义务教育阶段学杂费，补助困难家庭学生课本费和寄宿生生活费；城乡困难家庭学生救助做到全覆盖，并根据困难程度区别对待，提高标准，规范发放，救助资金由教育部门一个口筹集，一个漏斗发放，确保没有一个中小学生因贫困而辍学，确保考上大学的学生没有一个因贫困不能及时入学。二是各级政府负责购买公益岗位安置困难群众就业，确保零就业家庭至少一人就业，确保有劳动能力的残疾人家庭至少一人就业，确保特困家庭至少一人就业，确保双下岗“4050”家庭至少一人就业，优先保证特困家庭人员就业。三是通过政府补贴和社会救助，保证特困家庭、残疾人、孤儿、丧失劳动能力人员有房住、有饭吃、有衣穿、有病能就医。四是完善市县社会福利院，完成茄子河和新兴区社会福利院建设，年底前达到城镇“三无”人员和农村五保户集中供养条件。五是完成城乡困难家庭白内障患者免费复明手术，分区建设残疾人康复中心。六是为城镇低保户提供供热、供电补贴。七是市区分别启动城区廉租房建设。八是各级政府组合司法部门，无偿为困难群众提供法律援助。九是精心组织、科学安排，有效地开展“一帮一”解困活动。十是办好城镇慈善超市，为困难群众提供急需生活用品。

　　 （九）全力做好安全稳定工作，创建平安七台河。

　　 一是突出抓好安全生产。严格按国家和省政策规定，做好整顿关闭矿井工作。严格按安全生产标准，加强安全监管、行业管理和技术指导。严格落实企业安全主体责任和有关部门监管责任，对发生三人以上事故的责任领导、监管人员和矿主从严处理。坚决关闭不符合安全生产条件矿井和打击非法井，坚决杜绝三人以上重特大事故，减少一般性事故。加强交通安全专项整治，坚决遏制事故高发势头。搞好重大火灾隐患整改，防止重特大火灾和群死群伤事故发生。抓好森林防火，实现26年无森林火灾。加大食品、药品市场监察力度，加强易燃、易爆、危险化学品监管，确保公共安全。二是继续推进“平安七台河”创建活动。进一步加强社会治安综合治理，重点开展侦破命案专项行动、打击侵财犯罪和打黑除恶专项斗争。推进“三基”工程，提高政法队伍整体素质，确保公正文明执法。坚决打击“法轮功”等邪教组织和境内外敌对势力破坏活动，确保社会政治稳定。进一步完善突发公共事件预案，提高对重大安全事故及突发事件的快速动员和应急保障能力。三是进一步做好信访工作。认真贯彻《信访条例》，坚持分级负责、归口办理和谁主管、谁负责的原则，发挥各级信访联席会议作用，组合各类资源处理信访问题。进一步明确各级领导信访责任制，坚持领导信访接待日制度。加大依法治访力度，引导群众依法有序表达诉求。

　　 （十）深入推进各项改革，进一步增强发展活力。

　　 坚持市场经济的改革取向，全面深化经济社会各个领域的改革，为落实科学发展观和构建和谐社会提供体制机制保障。一是积极稳妥推进事业单位改革。依据全省事业单位改革方案，坚持分类指导，有序推进，严格掌握政策，做好科学分类、资源整合的基础工作，按照省统一部署启动事业单位改革。二是继续推进城市和农村信用社改革。推进城市信用社单一法人社组建工作，力争一季度挂牌运营，加快向城市商业银行过渡。加强对农村信用社改革试点相关考核指标的监测，保证市、县联社在六月末前达到“出口”标准，争取央行票据兑付一次成功，年内完成统一法人社组建。三是深入推进农村综合改革。巩固农村税费改革成果，确保农民负担不反弹。坚持涉农补贴资金“一折通”发放，确保及时足额兑付到农民手中。做好村级债务调查摸底工作，研究化解陈欠债务措施，严格控制新生债务。按照“依法、自愿、有偿”的原则，加快农村土地流转。四是继续向区级放权。按照完善功能、扩大职能需要，继续向区级下放权力，赋予区级政府更多抓发展的自主权。

　　 四、进一步加强政府自身建设

　　 新的形势和任务对政府工作提出了新的更高的要求，九届政府必须适应落实科学发展观和构建和谐社会的要求，不断加强自身建设，努力做到为人民服务、对人民负责、受人民监督、让人民满意。一是坚持转变职能，建设服务政府。继续推进行政管理体制改革，更加注重履行社会管理和公共服务职能，淡化权力和管制观念，增强责任和服务意识，把工作着力点放在发展社会事业、创造发展环境、提高公共服务水平和构建和谐社会上。深入贯彻《行政许可法》，进一步清理和规范行政审批项目，理顺部门分工，强化三区政府职能，提高行政效能。创新政府管理方式，推进电子政务建设。加强公务员学习培训，完善考试录用制度，健全考核评价体系，提高落实科学发展观的能力，构建和谐社会的能力，抓落实解难题的能力。二是坚持依法行政，建设法治政府。依法规范行政行为，健全和完善行政权力监督机制，自觉接受人大的依法监督和政协的民主监督。大力推进政务公开，完善公开制度，拓宽公开领域，保障人民群众的知情权、参与权和监督权。进一步完善行政决策机制，健全集体领导和集体决策制度，对专业性、技术性较强和投资较大的项目，实行专家论证、技术咨询和决策评估，涉及群众利益的重大决策，严格按规定程序公示或听证，提高政府公信力。三是坚持以人为本，建设为民政府。牢固树立执政为民理念，切实把实现好、维护好、发展好人民群众的根本利益作为政府工作的出发点和落脚点，经常深入群众体察民情、了解民意，集中财力优先解决涉及群众利益的就学、就医、就业、住房等问题。认真纠正损害群众利益的不正之风，解决好公共服务不到位、执法不公等群众反映强烈的问题。四是坚持转变作风，建设务实政府。把求真务实、创新高效的要求落实到政府工作的各个环节，一心一意谋发展，集中精力抓落实。坚持讲实话、重实干、办实事、求实效，力戒形式主义，不做表面文章，防止急功近利。大力精简会议和文件，减少不必要的应酬和事务性活动。倡导各级干部多深入企业、社区和农村，帮助群众解决实际问题。五是坚持从严治政，建设廉洁政府。政府组成人员带头搞好廉洁自律，严格管好亲友和身边工作人员。扎实开展以工程建设、土地出让、政府采购、国有资产处置等领域为重点的治理商业贿赂工作。加大对部门私设小金库、挪用专项经费、滥发补助和福利、公款旅游等问题的审计监察力度。严厉查处办事要好处、收费罚款不开收据等损害企业和群众利益的行为。加强对行政审批等权力运行的监督，落实好重要岗位人员轮岗、领导干部离任审计等制度，真正做到用制度管权、按制度办事、靠制度管人，不断开创政府廉政建设新局面。

　　 各位代表，新的五年向我们展示了建设龙江强市的美好前景，新一届政府的使命光荣而艰巨。我们坚信，有省委、省政府和市委的正确领导，有人大政协的监督支持，有全市人民的团结奋斗，我们一定会在全面建设小康社会和构建和谐七台河的伟大征程中迈出更大的步伐，建设龙江强市的宏伟目标一定能够实现。
